# Histopathology Findings of Low‐Level Laser Therapy Effectiveness on Achilles Tendon Repair in Rabbit Model

**DOI:** 10.1002/vms3.70347

**Published:** 2025-04-11

**Authors:** Nima Najafi Tabrizi, Mehdi Marjani, Vooria Tohidi, Zohreh Ghorannevis

**Affiliations:** ^1^ Department of Surgery, Karaj Branch Islamic Azad University Karaj Iran; ^2^ Department of Clinical Sciences, Science and Research Branch Islamic Azad University Tehran Iran; ^3^ Department of Radiology, Karaj Branch Islamic Azad University Karaj Iran; ^4^ Department of Physics, Karaj Branch Islamic Azad University Karaj Iran

**Keywords:** histopathology finding, Immunohistochemistry findings, low‐level laser therapy, rabbit, tendon repair

## Abstract

**Background:**

Low‐level laser therapy (LLLT) has been utilized to treat tendinitis and various other musculoskeletal conditions. The current study assessed the impact of LLLT (650 and 750 nm) on tendon repair in rabbits.

**Materials and methods:**

Fifteen 2‐year‐old male New Zealand White rabbits were divided into three groups: control, 650 nm laser and 750 nm laser therapy. After applying Achilles tendon‐destructive surgery on their right legs, rabbits underwent LLLT, and tendon repair was assessed using histopathology and immunohistochemistry (IHC) findings. All data were analysed using SPSS version 21, considering a significant level <0.05.

**Results:**

The study's histopathological and immunohistochemical analysis revealed that LLLT at 650 and 750 nm significantly improved tendon healing compared to the control group (*p* < 0.05). The treated groups exhibited better organized tendon fibres with reduced discontinuity, collagen fibre waviness, and inflammatory response (*p* < 0.05). Both laser wavelengths showed similar results with no significant differences between them (*p* > 0.05), but both were notably better than the control group in reducing inflammation, enhancing fibre structure, and lowering levels of collagen type I (Col‐I); collagen type III (Col‐III); transformer growth factor beta (TGF‐β); galectin‐3 (galectin‐3); VGF nerve growth factor inducible; vascular endothelial growth factor (VEGF), indicating a more effective healing process with LLLT.

**Conclusion:**

Due to our findings, LLLT at 650 and 750 nm effectively reduced inflammation, improved structural integrity, and enhanced the organization of collagen fibres for Achilles tendon repair in rabbits.

## Graphical abstract

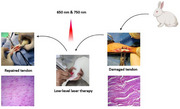

**Summary**

- **Enhanced tendon healing**: Low‐level laser therapy (LLLT) at both 650 and 750 nm significantly improved Achilles tendon repair, reduced collagen fibre waviness and lower inflammatory response.
- **Modulation of key biomarkers**: LLLT resulted in decreased levels of Col‐I, Col‐III, TGF‐β, galectin‐3, VGF and VEGF, indicating a more effective healing process with reduced fibrosis and enhanced tissue regeneration.
- **Comparable efficacy of both wavelengths**: Both 650 and 750 nm laser therapy were equally effective in promoting Achilles tendon healing.

## 1 Introduction

Tendon healing is a slow repair process that results in poorly healed tissue that often cannot regain full range of motion. The initial stages of tendon healing involve forming a weakly active tissue incapable of withstanding the tensile stresses necessary for joint movement (Brumitt 2021; Nichols et al. 2019). Immobilizing the limb can expedite healing but leads to adhesions between the tendon and its sheath, reducing the tendon's sliding ability within the sheath (Liu et al. 2018; Liu et al. 2022). Applying pressure during healing is essential to prevent these adhesions but carries the risk of tendon fibre rupture (Capella‐Monsonís et al. 2019; Li et al. 2021). The main goals in managing acute Achilles tendon rupture are to accelerate the permanent healing of the tendon to its correct length and size and to ensure the rapid recovery of normal tendon function (Connizzo et al. 2014).

Tendon repair involves three overlapping stages: inflammation, proliferation, and remodelling. The inflammatory phase initiates healing through immune cell infiltration, releasing cytokines to clear debris, and promotes angiogenesis (Darrieutort‐Laffite et al. 2024; Yang et al. 2021). In the proliferation stage, fibroblasts and tenocytes synthesize extracellular matrix components, such as collagen, to restore structural integrity. Finally, the remodelling phase organizes collagen into bundles, improving tensile strength, although the repaired tendon often remains biomechanically inferior to the original (Yang et al. 2021; Citro et al. 2023). This process requires a fine balance between immune response and mechanical forces to optimize regeneration and restore tendon functionality.

The ideal treatment method for tendon rupture should be quick, feasible and cost‐effective, with fewer complications or abnormalities post‐operation (Park et al. 2020). Surgical intervention is the most prevalent for tendon rupture among the common treatments. However, numerous complications have been reported following surgery, such as adhesion to surrounding connective tissues and poor repair properties, often resulting in unsatisfactory outcomes (Subaşı et al. 2023; Wang et al. 2024). Treatment protocols after surgery include the use of nonsteroidal anti‐inflammatory drugs (NSAIDs) to reduce pain and inflammation (Lisboa et al. 2017; Meunier and Larrey 2018). However, the use of NSAIDs has been associated with spontaneous tendon destruction, and several studies have raised concerns about their severe side effects, including liver toxicity, kidney toxicity, coagulation problems, heart issues, and damage to the digestive system mucosa (Zhao‐Fleming et al. 2018).

Recently, laser therapy has shown promising results in tendon healing, particularly for the Achilles tendon (Nogueira and Júnior 2015; Lyu et al. 2022). Low‐level lasers with a power not exceeding 500 mW have been found to reduce the inflammatory phase's duration and promote the alignment of collagen fibres longitudinally along the tendon (Barbosa et al. 2013; Marcos et al. 2014). Low‐level laser treatment has been reported as a non‐invasive, non‐damaging, non‐carcinogenic treatment method with no significant side effects in many conditions. However, low‐level lasers' parameters and mechanisms of action are not fully understood (Hamblin et al. 2016; Mussttaf et al. 2019). Utilizing a safe method to accelerate tendon healing is crucial to prevent complications from prolonged inflammation and immobilization. Therefore, in the current study, we investigated the effectiveness of low‐level laser therapy (LLLT) (650 and 750 nm) on tendon repair in rabbits.

## 2 Materials and Methods

### 2.1 Preparation of Animal

Fifteen 2‐year‐old male New Zealand White rabbits with a mean weight of 2470 g were included in the present study. Rabbits were randomly divided into control, 650, and 750 nm laser therapy groups (*n* = 5 in each group). The rabbits were individually housed in cages and provided with appropriate pellet food (high in fibre and low in protein) twice daily, along with access to fresh water. They were kept at a temperature of 24°C with a humidity level of 20%–30%.

### 2.2 Surgical Procedure

Before the surgery, the rabbits underwent 12 h of food abstinence and two hours of water abstinence to prevent complications from anaesthesia. For the surgical procedure, the right leg of each rabbit was shaved and scrubbed. Xylazine 2% and Ketamine 10% (Alfasan, Netherlands; Sir Aldawa Co., Iraq) were administered at a dose of 5 mg/kg and 35 mg/kg of body weight, respectively. The same for all administered products (Tranquilli et al. 2013). If additional anaesthesia was required during the operation, half of the initial dose was prepared in separate syringes for injection as needed.

Fluid therapy (sodium chloride) was administered at 10 mg/kg of body weight to maintain the animals’ body fluid balance, and a betadine surgical scrub was done. To expose the right Achilles tendon, an incision was made on the lateral side, parallel to the tendon, extending from 1 cm above the calcaneus bone to 2 cm below the gastrocnemius muscle belly (total length: 2 cm). Following the dissection and separation of the tendon from the surrounding tissues, the Achilles tendon was successfully exposed. Subsequently, to induce tendinopathy, 10 longitudinal scratches were made along the tendon using an 11‐blade scalpel. After confirming the creation of the longitudinal scratches, the skin was sutured. Due to the poor nerve and blood supply in the distal Achilles tendon, the proven effects of LLLT in reducing pain and inflammation, the absence of pain symptoms in the rabbits and the delayed effect of analgesic drugs on tendon healing, analgesics were not used after irritation was induced in the rabbits.

### 2.3 Laser Therapy

A low‐level laser (GaAIAs, Class 3B, 650 and 750 nm, 200 mW, spot size = 0.196 cm^2^, power density = 1.020 W/cm^2^, irradiation time = 30 s, total dose = 54 J) was used for laser therapy at 650 and 750 nm levels. The day following surgery, rabbits were transferred to the operating room for laser therapy. Laser therapy was administered once a day for four sessions, followed by one week of rest (Days 1, 8, 15, 22, 29, 36, 42). During these sessions (42 days), laser therapy operations were performed on the affected limbs, in both lateral and sagittal aspects, with each area receiving treatment for 10 s.

### 2.4 Histopathology and Immunohistochemistry (IHC) Evaluation

After 42 days, rabbits were returned to the operating room for tendon sampling. Injured tendons from the 650 nm laser and 750 nm laser groups, along with a healthy intact tendon as a reference for normal tissue, were excised for histopathological and IHC evaluations. The samples were fixed in 10% neutral‐buffered formalin for 24–48 h, dehydrated using graded ethanol, embedded in paraffin, and sectioned at 4–5 µm thickness. Haematoxylin and eosin (H&E) and Masson's trichrome staining were performed to assess fibre alignment, inflammatory changes, neovascularization, cellular density and nuclear roundness.

For IHC analysis, sections underwent antigen retrieval in citrate buffer (pH 6.0) at 95°C for 20 min using a water bath, followed by blocking with 5% bovine serum albumin (BSA) for 30 min at room temperature. Primary antibodies targeting collagen type I (Col‐I), collagen type III (Col‐III), transforming growth factor beta (TGF‐β), galectin‐3, VGF nerve growth factor inducible (VGF), and vascular endothelial growth factor (VEGF) were applied at appropriate dilutions and incubated overnight at 4°C. The samples were then treated with an HRP‐conjugated secondary antibody for 30 min, followed by DAB chromogen application and counterstaining with haematoxylin.

### 2.5 Statistical Analysis

Data were presented as mean ± standard error (SE) and percentages. Tukey's multiple comparisons test was performed to compare the results between groups. Data were analysed using SPSS software version 21. A significant level was set at 0.05.

## 3 Results

### 3.1 Histopathologic Findings

**FIGURE 1:**
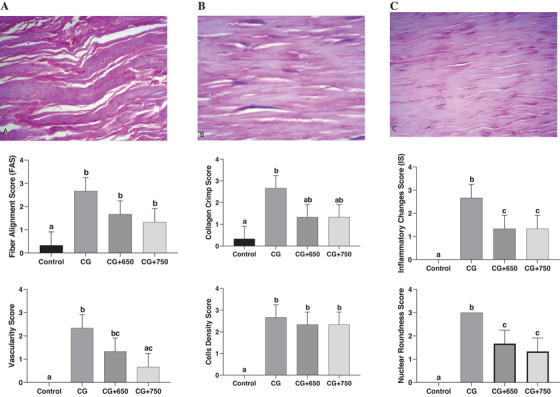
Microscopic view of the structure and arrangement of collagen fibres in the Achilles tendon of rabbits. (A) Control group, (B) 650 nm laser group, (C) 750 nm laser group. The images were captured at a scale of 10 µm using a 40× lens, 50 µm using a 10× lens. After 42 days, the collagen fibre structure in the control group was significantly disrupted, with strands appearing discontinuous and wavy. In contrast, the 650 and 750 nm laser groups exhibited only slight interruptions, with a reduction in strand length and wavelength.

Microscopic findings of the control group on Day 42 regarding the tendon fibre structure demonstrated a significant discontinuity in fibre structure in the control group (2.66 ± 0.57), which was less evident in the 650 nm laser (1.66 ± 0.57) and the 750 (1.33 ± 0.57) nm laser groups. The collagen fibre arrangement in the control group on Day 42 showed a cross‐linked and wavy pattern. In contrast, the 650 and 750 nm laser groups displayed slightly reduced collagen fibre length and waviness (Figure 1).

The 650 and 750 nm laser groups showed a more organized fibre structure with less discontinuity, reduced fibre length, and less waviness. There was no significant difference in fibre structure and arrangement between the 650 and 750 nm laser groups (*p* > 0.05). In the evaluation of the inflammatory response to a tendon injury, both the 650 nm (1.33 ± 0.57) and 750 nm (1.33 ± 0.57) laser groups exhibited a significantly reduced inflammatory response compared to the control group (2.66 ± 0.57) (*p* < 0.05). No significant difference in the inflammatory response was observed between the 650 and 750 nm laser groups (Table 1).

**TABLE 1:** Histopathology findings of the Achilles tendon repair of rabbits in control, 650 and 750 nm laser therapy groups.

Histopathology evaluation		Mean differences	95% CI Min–Max	Adjusted *p* value^a^
**Fibre alignment**	Laser 650 vs. control	−1.00	−2.510 to 0.5096	0.225
	Laser 750 vs. control	−1.33	−2.843 to 0.1763	0.084
	Laser 650 Vs. laser 750	−0.33	−1.843 to 1.176	0.891
**Inflammatory changes**	Laser 650 vs. control	−1.33	−2.64 to −0.02	0.045
	Laser 750 vs. control	−1.33	−2.64 to −0.02	0.045
	Laser 650 vs. Laser 750	0.00	−1.30 to −1.30	>0.999
**Neovascularization**	Laser 650 vs. control	−1.00	−2.30 to 0.30	0.144
	Laser 750 vs. control	−1.66	−2.97 to 0.35	0.014
	Laser 650 vs. Laser 750	−0.66	−1.97 to 0.64	0.414
**Cellular density**	Laser 650 vs. control	−0.33	−1.64 to 0.97	0.845
	Laser 750 vs. control	−0.33	−1.64 to 0.97	0.845
	Laser 650 vs. laser 750	0.00	−1.30 to 1.30	>0.999
**Nuclear roundness**	Laser 650 vs. control	−1.33	−2.40 to 0.26	0.016
	Laser 750 vs. control	−1.66	−2.73 to −0.59	0.004
	Laser 650 vs. Laser 750	−0.33	−1.40 to 0.73	0.753

^a^ Tukey's multiple comparisons test.

The vascular changes and angiogenesis evaluation showed a 30% increase in vascularization in the control group (2.33 ± 0.57). In the 650 nm (1.33 ± 0.57) and 750 nm (0.66 ± 0.57) laser groups, a 20% increase in angiogenesis was observed. No statistically significant difference was found between the control and 650 nm laser groups (*p* > 0.05). However, a significant difference (*p* < 0.05) was observed between the control and 750 nm laser groups. The cellular density showed no statistically significant differences among the control (2.66 ± 0.57), 650 nm laser (2.33 ± 0.57), and 750 nm laser (2.33 ± 0.57) groups (*p* > 0.05). Each group exhibited a moderate increase in cellular density. Nuclear structure demonstrated significant differences between the 650 nm laser (1.66 ± 0.57), 750 nm laser (1.33 ± 0.57), and the control group (3.00 ± 0.00) (*p* < 0.05). The nuclei in the control group were highly rounded, whereas in the 650 and 750 nm laser groups, the nuclei were only slightly rounded (Table 1).

### 3.2 IHC Findings

**FIGURE 2:**
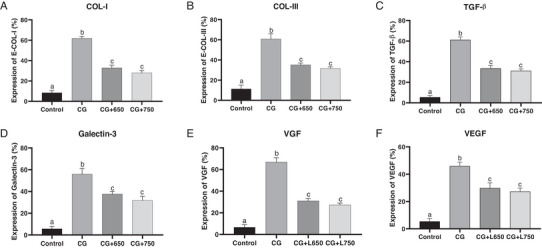
Effectiveness of Achilles tendon repair of rabbits in 650 and 750 nm laser therapy groups compared to the control group. The immunohistochemistry (IHC) findings revealed significant differences between the control group and both laser therapy groups (650 and 750 nm) across all evaluated markers. *Tukey's multiple comparisons test; adjusted *p* < 0.05 with 95% CI. A) For Col‐I, both laser therapy groups showed significantly higher levels compared to the control, with mean differences of 28.93 (22.88–34.99) for 650 nm and 33.89 (27.83–39.94) for 750 nm (*p* < 0.0001), and no significant difference was observed between the two laser groups (*p* = 0.1139). B) For Col‐III, both laser groups exhibited significantly higher levels than the control, with mean differences of 25.6 (16.64–34.55) and 29.1 (20.14–38.05) for the 650 and 750 nm groups, respectively (*p* < 0.0001), whereas the difference between the laser groups was not significant (*p* = 0.6148). C) TGF‐β levels were significantly elevated in both laser‐treated groups compared to the control, with mean differences of 27.74 (21.42–34.06) and 30.15 (23.83–36.47) for the 650 and 750 nm lasers, respectively (*p* < 0.0001), with no significant inter‐laser group difference (*p* = 0.6319). D) Galectin‐3 levels were also significantly higher in both laser groups compared to the control, with mean differences of 18.36 (9.085–27.64, *p* = 0.001) for 650 nm and 24.22 (14.94–33.49, *p* = 0.0001) for 750 nm, though the difference between the laser groups was not statistically significant (*p* = 0.2572). E) For VGF, the laser groups showed significant increases compared to the control (*p* < 0.0001), with mean differences of 35.9 (28.77–43.03) and 39.64 (32.51–46.76) for the 650 and 750 nm groups, respectively, but no significant difference was observed between the laser groups (*p* = 0.3927). F) VEGF was significantly higher in the laser groups compared to the control, with mean differences of 16.14 (8.388–23.89, *p* = 0.0007) for 650 nm and 18.56 (10.81–26.31, *p* = 0.0003) for 750 nm. The difference between the two laser groups for VEGF was not significant (*p* = 0.7532). Col‐I, collagen type I; Col‐III, collagen type III; TGF‐β, transformer growth factor beta; VEGF, vascular endothelial growth factor; VGF, VGF nerve growth factor inducible.

The IHC analysis revealed a significant difference in Col I between the control (61.83 ± 1.96), 650 nm laser (32.90 ± 2.58), and 750 nm laser (27.94 ± 2.32) groups. Col I levels in the 650 and 750 nm laser groups were significantly lower compared to the control group (*p* < 0.001). No statistically significant difference in Col I levels was observed between the 650 and 750 nm laser groups (*p* > 0.05). The mean level of Col III was 60.76 ± 5.17, 35.16 ± 1.72, and 31.66 ± 1.73 in the control group, 650 nm laser group and 750 nm laser group, respectively. Col III level was significantly lower in the 650 and 750 nm laser groups compared to the control group (*p* < 0.001). No significant difference was observed between the two laser groups (*p* > 0.05). The levels of TGF‐β were significantly lower in the 650 nm laser (33.43 ± 2.86) and the 750 nm laser (31.02 ± 23.04) groups compared to the control group (61.17 ± 2.90) (*p* < 0.001). No significant difference was observed between the two laser groups (*p* > 0.05) (Figure 2).

**FIGURE 3:**
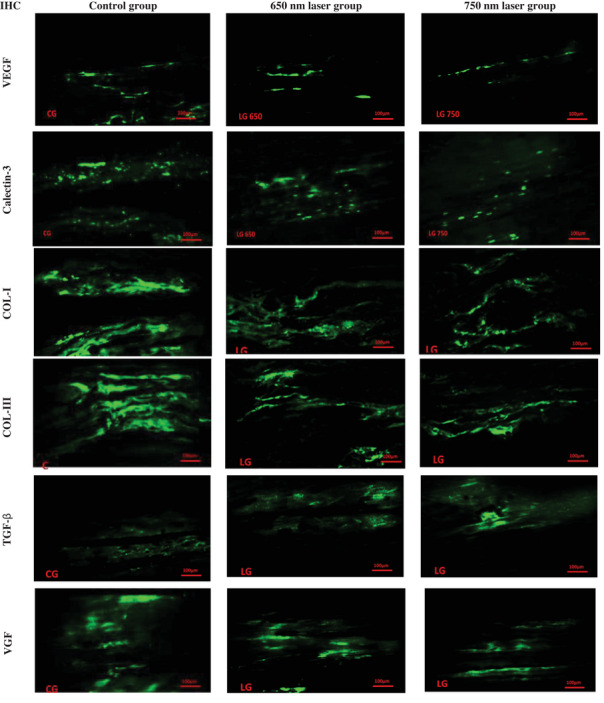
Immunohistochemistry (IHC) findings of Achilles tendon repair of rabbits in control, 650 and 750 nm laser therapy groups of rabbits. The images were captured at a scale of 100 µm using a 20× lens. Col‐I, collagen type I; Col‐III, collagen type III; TGF‐β, transformer growth factor beta; VEGF, vascular endothelial growth factor; VGF, VGF nerve growth factor inducible.

Galectin‐3 levels in 650 nm laser, 750 nm laser and control groups were 37.63 ± 2.39, 31.77 ± 3.73 and 55.99 ± 5.12, respectively. Galectin‐3 levels were significantly lower in the 650 and 750 nm laser groups compared to the control group (*p* = 0.001), and no significant difference was observed between the 650 and 750 nm laser groups (*p* > 0.05). The mean levels of VGF in control, 650 nm laser and 750 nm laser groups were 66.91 ± 3.96, 31.01 ± 2.18 and 27.27 ± 1.78, respectively. The VGF levels were significantly lower in the 650 and 750 nm laser groups compared to the control group (*p* < 0.001). There was no significant difference between the 650 and 750 nm laser groups (*p* > 0.05). VEGF levels were 45.86 ± 2.97, 29.72 ± 3.88 and 27.29 ± 2.59, in the control, 650 and 750 nm laser groups, respectively. VEGF levels were significantly lower in the 650 and 750 nm laser groups compared to the control group (*p* < 0.001) (Figures 2 and 3).

## 4 Discussion

Laser therapy has emerged as a promising modality for enhancing tendon repair because it modulates cellular activity, reduces inflammation, and promotes tissue regeneration in different models (Ning et al. 2023; Liang et al. 2024). LLLT expands therapeutic modalities within veterinary medicine for addressing inflammatory conditions, facilitating analgesia, and fostering the repair of damaged tissues across a spectrum of conventional and non‐traditional animal species (Paterniani and Grolli 2018). LLLT utilizes near‐infrared light to induce non‐thermal and photochemical reactions within cells resulted in fewer complications. Studies suggested that combining LLLT with specific exercise therapies can effectively treat tendinopathy (Lyu et al. 2022). Its non‐invasive nature and ability to mitigate inflammation and promote collagen synthesis make LLLT a promising modality for veterinary tendon repair (Wickenheisser et al. 2019; Girgis and Duarte 2020).

Compared to the control group, we found the tendon healing process acceleration in groups treated with 650 and 750 nm laser. The microscopic view of a healthy tendon showed long, continuous fibres arranged parallel and densely packed, which revealed that the structure contrasted significantly with the control, 650 and 750 nm laser groups. Da Ré Guerra et al. (2017) reported that LLLT effectively reduces non‐collagenous protein and glycosaminoglycan content while increasing metalloproteinase‐9 levels in inflamed tendons, demonstrating its potential to restore balance in inflamed tissues within four hours of treatment. Carrinho et al. (2006) demonstrated that LLLT was effective for tendon repair in mouse models, particularly at 685 nm. Naterstad et al. (2018) reported that LLLT prevented bleeding and reduced inflammation severity better than other treatments. Evidence demonstrated the accelerated role of LLLT on histological, physiological and biomechanical tendon healing in animal models (He et al. 2023).

The histopathological and IHC results of the present study revealed that on Day 42, the control group exhibited significant discontinuity in the tendon fibre structure. In contrast, the 650 and 750 nm laser groups displayed less disruption, indicating the beneficial effect of LLLT on tendon healing. Both laser groups (650 and 750 nm) showed more organized fibre structure, reduced inflammation and enhanced angiogenesis compared to the control group. Additionally, cellular density moderately increased in all groups, whereas nuclear structure showed significant differences, with the laser groups (650 and 750 nm) displaying less rounded nuclei than the control group.

A study by Allahverdi et al. reported that LLLT on tendon repair showed variations in inflammation, collagen arrangement, and adhesion formation among the groups, with LLLT expected to reduce inflammation, promote more regular collagen fibre arrangement and minimize adhesion formation compared to the control. However, this excerpt does not provide the exact outcomes (Allahverdi et al. 2015). Another study by Alkhilani et al. demonstrated that diode laser therapy at 904 nm, particularly in impulse mode, significantly enhanced tendon healing by improving inflammatory and fibroblast responses and promoting better collagen differentiation during the remodelling phase. Impulse diode laser was found to be more effective than continuous laser therapy, which showed sustained high cellular responses but less organized collagen fibres (Alkhilani and Atta 2020).

Similarly, our study found that LLLT at 650 and 750 nm on tendons exhibited reduced inflammation, better‐organized collagen fibres, and improved histopathological and immunohistochemical markers. Both wavelengths were equally effective, reinforcing the role of laser therapy in promoting tendon healing. A study by Lyu et al. illustrated that LLLT aids tendon repair by promoting angiogenesis, collagen synthesis and anti‐inflammatory responses across healing phases. However, excessive growth factor upregulation may lead to fibrosis, requiring further research to optimize its use (Lyu et al. 2022). Moreover, we observed that Col I, Col III, TGF‐β, galectin‐3, VGF and VEGF levels were significantly lower in both laser groups (650 and 750 nm) compared to the control group, suggesting that LLLT contributes to reduced inflammation.

A study by Torres‐Silva et al. illustrated that the low‐level laser (at 660 nm) applied to treat collagenase‐induced tendinitis in rat Achilles tendons effectively reduced significant pro‐inflammatory markers (Torres‐Silva et al. 2015). Haslerud et al. (2017) reported that combining cryotherapy with LLLT can yield an additional anti‐inflammatory effect, such as reducing levels of pro‐inflammatory markers in the acute phase of tendon injury. A study by Laraia et al. (2012) found that LLLT (660 nm) significantly reduced inflammatory cytokines while increasing anti‐inflammatory cytokines in injured rat Achilles tendons, suggesting its role in modulating inflammatory cytokine release and promoting healing. Although the current study provided valuable insights into the effectiveness of LLLT (650 and 750 nm), further research is warranted to fully elucidate its mechanisms of action and optimize treatment protocols for clinical application in veterinary medicine.

## 5 Conclusion

The current study's findings demonstrated that LLLT's efficacy (650 and 750 nm) in enhancing tendon repair and reducing inflammation is complemented by its non‐invasive nature and minimal risk profile. However, continued monitoring and research are necessary to further validate its safety and efficacy across various animal species and clinical scenarios.

## Author Contributions

Mehdi Marjani, Nima Najafi Tabrizi and Vooria Tohidi participated in the research design. Nima Najafi Tabrizi, Zohreh Ghorannevis and Vooria Tohidi participated in writing the first draft. Mehdi Marjani and Zohreh Ghorannevis participated in the performance of the research and analytic tools. Zohreh Ghorannevis participated in data analysis. All authors reviewed and confirmed the final manuscript.

## Ethics Statement

This study was approved by the ethics committees of the Karaj branch, Islamic Azad University [IR.IAU.K.REC.1401.061]. Animal handling and testing were performed according to the EU ethical guidelines for animal testing (2010/63/EU).

## Consent

The authors have nothing to report.

## Conflicts of Interest

The authors declare no conflicts of interest.

## Declaration of Generative AI in Scientific Writing

While preparing this work, the authors used ChatGPT to improve the manuscript's language and edit the grammatical errors. After using this tool, the authors reviewed and edited the content as needed.

### Peer Review

The peer review history for this article is available at https://www.webofscience.com/api/gateway/wos/peer‐review/10.1002/vms3.70347.

## Data Availability

The study protocol and the datasets analysed are available from the corresponding author upon request.
